# The HIV care continuum among resident and non‐resident populations found in venues in East Africa cross‐border areas

**DOI:** 10.1002/jia2.25226

**Published:** 2019-01-24

**Authors:** Jessie K Edwards, Peter Arimi, Freddie Ssengooba, Grace Mulholland, Milissa Markiewicz, Elizabeth A Bukusi, Judy T Orikiiriza, Arti Virkud, Sharon Weir

**Affiliations:** ^1^ Department of Epidemiology University of North Carolina at Chapel Hill Chapel Hill North Carolina USA; ^2^ U.S. Agency for International Development Kenya/East Africa Regional Mission Nairobi Kenya; ^3^ Makerere School of Public Health Kampala Uganda; ^4^ MEASURE Evaluation Carolina Population Center University of North Carolina at Chapel Hill Chapel Hill North Carolina USA; ^5^ Centre for Microbiology Research Kenya Medical Research Institute Nairobi Kenya; ^6^ Rwanda Military Hospital Kigali Rwanda; ^7^ Infectious Diseases Institute College of Health Sciences Makerere University Kampala Uganda

**Keywords:** Viral Load, border crossing, transients and migrants, sex workers, East Africa, HIV Care Continuum, viral suppression, linkage to care

## Abstract

**Introduction:**

HIV care and treatment in cross‐border areas in East Africa face challenges perhaps not seen to the same extent in other geographic areas, particularly for mobile and migrant populations. Here, we estimate the proportion of people with HIV found in these cross‐border areas in each stage of the HIV care and treatment cascade, including the proportion who knows their status, the proportion on treatment and the proportion virally suppressed.

**Methods:**

Participants (n = 11,410) working or socializing in public places in selected East Africa cross border areas were recruited between June 2016 and February 2017 using the Priorities for Local AIDS Control Efforts method and administered a behavioural survey and rapid HIV test. This approach was designed to recruit a stratified random sample of people found in public spaces or venues in each cross border area. For participants testing positive for HIV, viral load was measured from dried blood spots. The proportion in each step of the cascade was estimated using inverse probability weights to account for the sampling design and informative HIV test refusals. Estimates are reported separately for residents of the cross border areas and non‐residents found in those areas.

**Results:**

Overall, 43% of participants with HIV found in cross‐border areas knew their status, 87% of those participants were on antiretroviral therapy (ART), and 80% of participants on ART were virally suppressed. About 20% of people with HIV found in cross border areas were sampled outside their subdistrict or subcounty of residence. While both resident and non‐resident individuals who knew their status were likely to be on ART (85% and 96% respectively), people on ART recruited outside their area of residence were less likely to be suppressed (64% suppressed; 95% CI: 43, 81) compared to residents (84% suppressed; 95% CI: 75, 93).

**Conclusions:**

People living in or travelling through cross‐border areas may face barriers in learning their HIV status. Moreover, while non‐residents were more likely to be on treatment than residents, they were less likely to be suppressed, suggesting gaps in continuity of care for people in East Africa travelling outside their area of residence despite timely initiation of treatment.

## 1 Introduction

Expanding economic integration has increased cross‐border movement and trade in East Africa 1. Communities that straddle international borders, including towns along highway border crossings and communities that serve as landing sites for fishing vessels from multiple countries, are important mixing environments for transient and resident populations. Characteristics of these “cross‐border areas”, including the presence of and interaction with highly mobile populations 2, density of venues offering alcohol 3 and opportunities for sex on site, influx of individuals with disposable income 4 and market for transactional sex 5 have been linked to high levels of HIV transmission. However, traditional HIV prevention, care and treatment programmes, often designed for long‐term residents of local catchment areas, may not adequately serve the needs of people with HIV found in cross border areas.

Early treatment resulting in viral suppression is an important strategy to reduce transmission of HIV from people living with HIV to their HIV‐uninfected partners 6, 7, 8. The UNAIDS 90‐90‐90 targets are designed to minimize the proportion of the population with an unsuppressed viral load by ensuring that 90% of people living with HIV know their status, 90% of those who know their status are on antiretroviral therapy (ART), and 90% of those on ART are virally suppressed 9. Baseline results from the SEARCH Trial conducted in rural communities in East Africa indicate that, prior to the intervention, over 60% of people with HIV had been previously diagnosed, nearly 80% of those who knew their status were on ART, and over 80% of those on ART were virally suppressed 10. Furthermore, the SEARCH trial, along with HIV prevention trials in other regions (e.g. the PopART trial in Zambia and South Africa 11) have identified several promising strategies to increase access to HIV testing 12, 13, retention in care 14, ART initiation 13 and viral suppression through universal testing and treatment.

However, HIV care and treatment in cross‐border areas face many challenges not encountered to the same extent in other areas, including an influx of mobile and transient populations, patients lost to follow‐up across international borders, and patients presenting for treatment away from their home countries, which may use different treatment regimens 15, 16, 17. Taken together, these challenges imply that people with HIV who live, socialize and travel in cross‐border areas may not be optimally served by existing HIV care and treatment programmes predominantly administered through a country‐focused lens. Accordingly, progress towards the 90‐90‐90 goals across the East African region may be negated by programming gaps in cross‐border areas.

Here, we describe the characteristics of people with HIV and progress towards the 90‐90‐90 goals in select East Africa cross‐border areas. The East Africa Cross Border Integrated Health Study (CBIHS) offers a rare opportunity to assess outcomes along the HIV care continuum for people living, working and socializing in cross‐border areas, including highly mobile cross‐border priority populations who are typically excluded from traditional epidemiologic studies implemented using household‐based surveys. Because such groups likely access services differently and face distinct sets of health risk factors 18, 19 from “resident” populations (i.e. those with a primary residence at the cross‐border area), we estimate the HIV care continuum and progress towards the 90‐90‐90 targets separately for resident and non‐resident groups.

## 2 Methods

### 2.1 Study procedures

**Figure 1:**
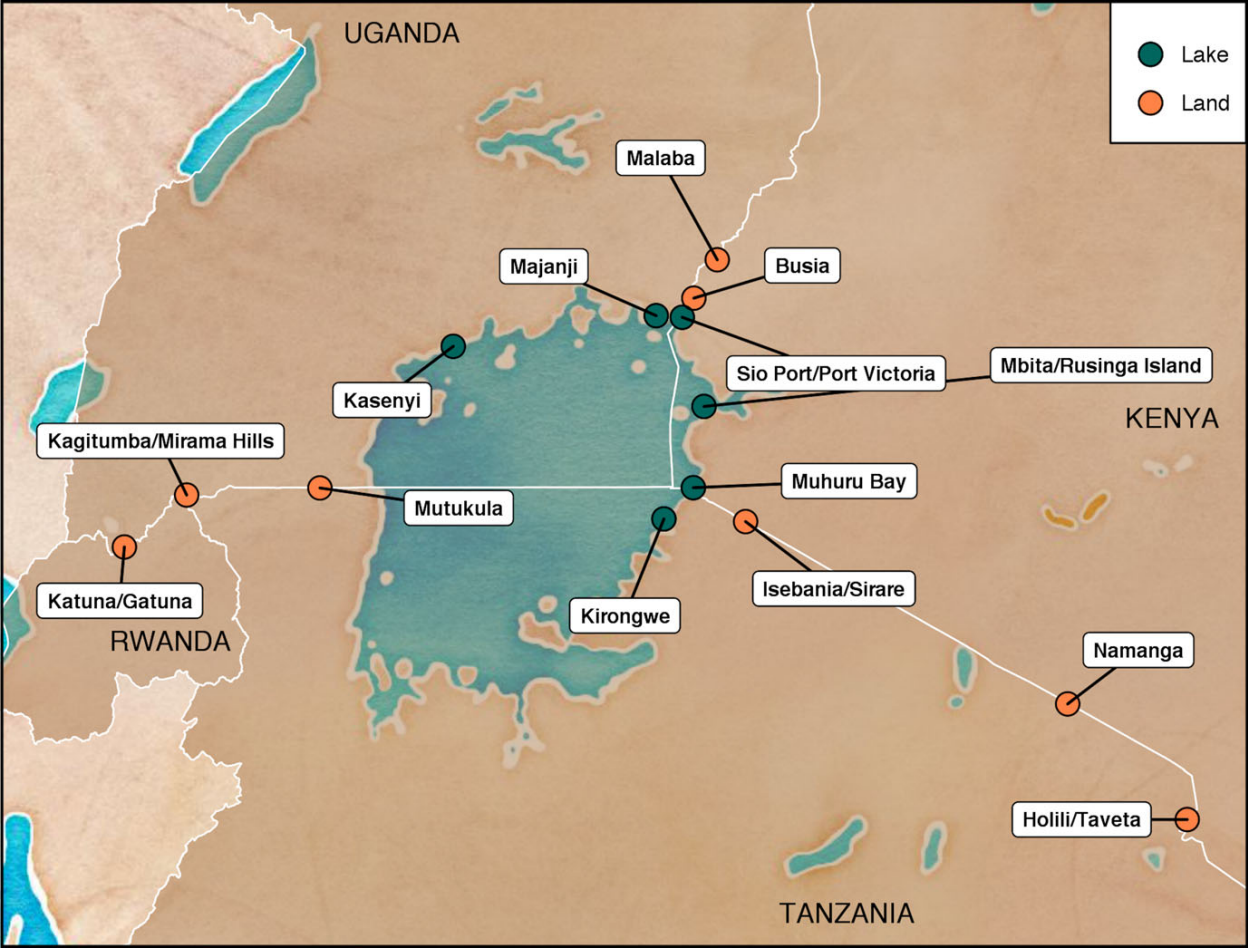
Map of land cross‐border areas (orange) and lake cross‐border areas (blue) in East Africa included in the East Africa Cross Border Integrated Health Study, 2016

The CBIHS is a population‐based cross‐sectional study of a wide array of health outcomes in 14 survey sites in cross‐border areas in Kenya, Uganda, Tanzania and Rwanda (Figure 1) conducted between June 2016 and February 2017. Of the selected sites, eight were “land border sites,” which included the area around international border posts on highways, and six were “lake border sites,” which included fishing villages on Lake Victoria that served as points of commerce for fisher folk from multiple East African countries. Land border sites included the area on both sides of the international border while lake sites contained area in a single country. All selected survey sites had a high level of cross‐border traffic and/or trade and sizeable populations.

As part of its mission to describe health outcomes and access to care, CBIHS conducted a bio‐behavioural survey among a sample of people patronizing or working in public venues in cross‐border areas. The Priorities for Local AIDS Control Efforts (PLACE) method 20, 21 was used to sample and recruit participants. The PLACE method consists of three steps designed to provide a stratified random sample of individuals socializing in cross border areas.

*Step 1*:* Enumeration of all venues in each cross border site:* Approximately 200 community informants in each cross‐border area were interviewed and each asked to provide the names and locations of up to 10 venues where people (including residents and non‐residents) socialize and meet new sexual partners. Additional community informants were interviewed until the list of venues reached saturation or no new venues were named.

*Step 2: Verification of a sample of venues*: The team verified characteristics of a sample of up to 100 venues in each cross‐border area to ensure that venues named in step 1 existed, were unique and were operational. In areas with fewer than 100 venues, all venues were included. In areas with more than 100 venues, a stratified random sample of venues was selected for verification.

*Step 3: Bio‐behavioural survey:* The team conducted a bio‐behavioural survey among individuals socializing at a stratified random sample of 40 venues per area. At sampled venues, a stratified sample of workers and male and female patrons was approached, with interview targets proportional to the total number of people at the venue. The appendix contains additional details on the sampling of venues and individuals.

Sampled individuals were offered counselling and rapid HIV testing according to the algorithm approved by each country. In addition, respondents were asked to participate in an interview to gather sociodemographic information, health history, family information, sexual behaviour, health‐seeking behaviour, and exposure to HIV prevention, care and treatment programmes, including ART. Participants who refused the HIV test were offered the option of participating in the interview. After the interview, participants who agreed to be tested received their result and post‐test counselling. Those with positive results were asked to provide dried blood spots for HIV‐1 RNA viral load testing, which was conducted according to national guidelines in each country. People newly testing positive for HIV were linked to care at a local health facility. Viral load measurements were communicated back to the facility with which the local staff who provided the HIV counselling and testing were associated. Respondents were given a card with an identification code and facility name so that they could obtain their viral load results. Participants who refused the HIV test were not invited to provide dried blood spots for viral load quantification.

To evaluate disparities in achieving the 90‐90‐90 goals between resident and non‐resident populations, we estimated the proportion in each stage separately for each group. We defined the “non‐resident” population as individuals who reported residing in a subnational administrative area (i.e. subdistrict, subcounty, parish, ward or commune) outside the cross border area where they were recruited. In contrast, the resident population consisted of individuals who reported that they reside within the subnational unit of the cross‐border area.

Study protocols were reviewed and approved by the University of North Carolina at Chapel Hill Institutional Review Board; Makerere University Higher Degrees, Research, and Ethics Committee in Uganda; the Kenya Medical Research Institute Ethics Review Committee; the National Institute for Medical Research in Tanzania; and the Rwanda National Ethics Committee. All participants in the biobehavioural survey provided written informed consent.

### 2.2 Statistical analysis

The purpose of this analysis was to estimate indicators related to the 90‐90‐90 targets in cross‐border areas, including:

1. The proportion of people living with HIV who know their status (the first 90)
2. The proportion of people who know that they are living with HIV who are on ART (the second 90)
3. The proportion of people on ART who have a suppressed viral load, defined as a viral load under 1000 copies/mL (the third 90); and
4. The overall proportion of people living with HIV who had a detectable viral load.

The questions used to ascertain whether an individual met the inclusion criteria for each step of the care continuum are provided in Table 1.

**Table 1:** Details on ascertainment of each step of the HIV care cascade

Step	Ascertainment algorithm	Instrument	Response
1. Knows status	A person living with HIV could have been considered to know his or her status in 2 ways:		
	1.1 Agree to the rapid HIV test and test positive; AND	Rapid HIV test	POSITIVE
	1.2 Report previously taking an HIV test; AND	SURVEY: Have you ever had an HIV test?	YES
	1.3 Report receiving the result and testing positive	SURVEY: Think back to the last test for which you collected your test result. What was the result? OR	INFECTED WITH HIV
		SELF‐COMPLETED: Think about the last timeyou were tested for HIV and got your test result. What was the result?	
	OR		
	2.1 Refuse the rapid HIV test; AND		MISSING
	2.2 Report previously taking an HIV test; AND	SURVEY: Have you ever had an HIV test?	YES
	2.3. Report receiving the result and testing positive	SURVEY: Think back to the last test for which you collected your test result. What was the result? OR	INFECTED WITH HIV
		SELF‐COMPLETED: Think about the last time you were tested for HIV and got your test result. What was the result?	
2. On ART	A person living with HIV was considered to be on treatment if he knew his status, as defined, above, and reported taking ART	SURVEY: Are you currently taking antiretroviral drugs (ART) to treat an HIV infection?	YES
3. Suppressed	A person living with HIV was considered to have a suppressed viral load if he or she		
	1. Agreed to provide a dried blood spot	Consent form	YES
	2. Viral load was below 1000 copies/mL	Dried blood spot	<1000 copies/mL

ART, antiretroviral therapy.

All participants who tested positive for HIV or who reported a previous positive HIV test were included. All analyses accounted for the survey design, including clustering by recruitment venue and stratified random sampling. Survey sampling weights were used to reweight the study sample to represent all people with HIV who socialize in public venues in the selected cross‐border areas, and we accounted for missing data due to informative refusals of the HIV test and viral load testing using inverse probability weights 22. Details on estimation of the weights can be found in the Appendix.

Each parameter (i.e. each 90‐90‐90 indicator) was estimated for the entire population and separately for resident and non‐resident populations. We also examined associations between achieving each stage of the HIV care continuum and other individual‐ and venue‐level characteristics using weighted bivariate (unadjusted) prevalence ratios (PRs). Corresponding 95% confidence intervals [CIs] were based on standard errors estimated using Taylor series linearization to account for the sampling design 23. Analyses were conducted in SAS 9.4 (Cary, NC). Because we aimed to quantify differences in outcomes between participants recruited within versus outside of their area of residence and not to evaluate a specific statistical hypothesis, no statistical hypothesis testing was performed 24.

## 3 Results

### 3.1 Description of the study sample

Of the 1,769 venues identified by community informants in the 14 cross‐border areas, 1,161 (66%) were sampled for verification. Of these sampled venues, 883 (76%) were successfully located, operational and contained a venue informant who consented to participate. A total of 452 of these venues were sampled for the bio‐behavioural survey, from which 11,567 individuals were sampled and asked to participate in the study. Of those, 11,410 (98.6%) agreed to participate in the interview and 10,549 (91.2%) agreed to be tested for HIV. Overall, 8656 (76%) of those completing the interview reported a place of residence within the cross border area and 2754 (24%) reported a place of residence outside the cross border area. Of those completing the interview, 576 individuals (5.0%) tested HIV positive or declined the HIV test and reported that they were HIV positive (460 residents and 116 non‐residents).

Of the 576 people living with HIV, 58% were female (n = 347), 68% were between the ages of 20 and 40 (n = 408), 69% had completed primary school or higher education (n = 411), and 16% reported receiving cash for sex in the past 12 months (n = 98) (Table 2). Approximately 20% of people with HIV found in the cross border areas reported a place (i.e. subdistrict or subcounty) of residence outside the cross border area. These non‐resident populations with HIV were younger, better educated and more likely to be female than resident populations with HIV. The majority of both resident and non‐resident populations reported spending fewer than two weeks per year away from their primary residence, but a notable proportion of both resident and non‐resident populations spent more than one month per year away from home (24% and 30% respectively).

**Table 2:** Characteristics of people living with HIV found at public venues in 14 cross‐border areas selected for the East Africa Cross Border Integrated Health Study, 2016

	Overall (N = 576)		Resident (n = 460)	Non‐resident (n = 116)
Characteristics	Sample *n*	Population %^a^	Population %	Population %
Gender				
Male	229	41.8	43.9	33.4
Female	347	58.2	56.1	66.6
Age				
15 to 19	21	3.8	2.7	8.2
20 to 29	210	35.2	34.5	38.1
30 to 39	198	32.9	32.8	33.3
40+	147	28.1	30	20.4
Employed	458	77.9	78.2	76.8
Paid cash for sex in past 12 months	76	12.0	12.4	10.1
Currently married or living with a partner	290	51.4	54.2	39.9
Education				
Less than primary	163	30.9	31.4	28.8
Completed primary	327	57.1	58.0	53.2
Completed secondary	54	7.4	5.6	14.3
More than secondary	30	4.7	5.0	3.7
Type of venue where recruited				
Bar/pub/restaurant	276	49.4	48.4	53.1
Hotel/guest house/lodge	117	19.6	19.9	18.5
Nightclub/disco/brothel	20	3.2	3.7	1.5
Commercial venue^b^	34	7.3	5.9	13
Outside venue^c^	57	10.2	11.7	4.2
Transportation hub^d^	5	0.6	0.4	1.5
Other	60	9.7	10.0	8.1
Time spent away from primary residence in past year				
Two weeks or less	339	60.0	60.0	60.2
More than two weeks but less than one month	86	14.9	16.1	10.3
More than one month but not more than three months	51	9.8	7.7	18.1
More than three months	68	10.5	11.3	7.6
Refused	28	4.7	4.9	3.9
Type of respondent				
Workers at venues	197	32.2	31.4	35.2
Patrons at venues	379	67.8	68.6	64.8
Recruited in a land border site	324	60.6	54.5	85.3
Recruited in a lake border site	252	39.4	45.5	14.7
Visited more than one venue on day of recruitment	254	46.0	46.0	45.8
Recruited at a venue where people have sex on site	316	49.6	48.0	51.8
Member of cross‐border priority population^e^				
Female sex worker (received cash for sex in past 12 months)	98	15.9	11.8	32.4
Fisher folk	94	14.5	15.6	10.0
Long distance truck driver	9	1.3	1.3	1.1
Female worker at venue	140	23.1	21.5	29.6
Young woman (ages 15 to 19)	95	16.0	14.3	23.0

^a^Population percentages were obtained by weighting the study sample to accommodate the complex sampling design; ^b^Commercial venues included markets, hair salons, shops, cinemas, recreation and game centres and schools; ^c^Outdoor venues included beaches, parks, construction sites and streets; ^d^Transportation hubs included truck stops and lorry/railway stations; ^e^Cross border priority populations were identified by local stakeholders and are not mutually exclusive

### 3.2 Progress towards the 90‐90‐90 indicators

**Figure 2:**
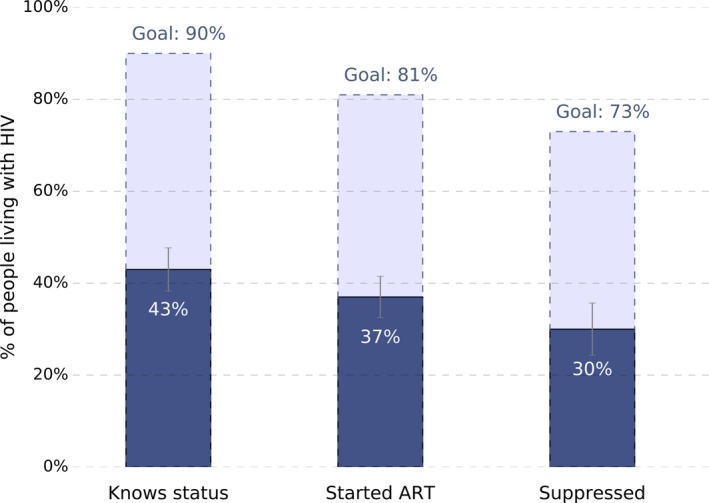
Marginal percentages of people living with HIV in each stage of the HIV care and treatment cascade and 95% confidence intervals in comparison to the 90‐90‐90 goals in the East Africa Cross Border Integrated Health Study, 2016

Among the 576 people living with HIV identified by the study, 270 already knew their status (weighted percentage: 43.0%; 95% CI: 38.2, 47.8) and 234 knew their status and were receiving ART (weighted percentage: 37.2%; 95% CI: 32.7, 41.8). Of those living with HIV, 29.5% knew their status, were receiving ART, and were virally suppressed (95% CI: 23.7, 35.2). These weighted percentages and 95% confidence intervals are compared visually to the 90‐90‐90 targets in Figure 2.

**Figure 3:**
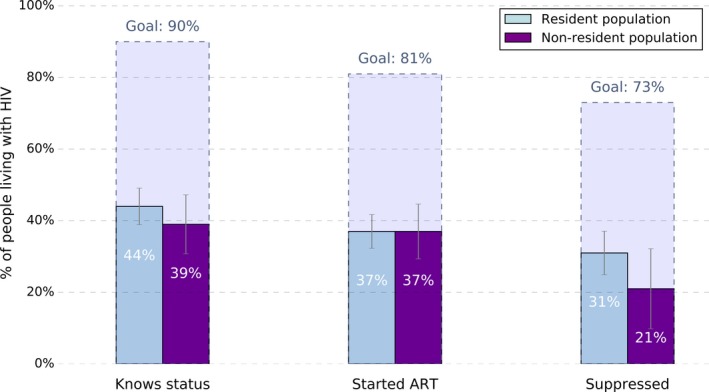
Marginal percentages of people living with HIV in each stage of the HIV care and treatment cascade and 95% confidence intervals in comparison to the 90‐90‐90 targets for resident and non‐resident populations in the East Africa Cross Border Integrated Health Study, 2016

Table 3 presents the weighted percentages of participants in each step of the cascade achieving the subsequent step overall and for resident and non‐resident subgroups. For example, among the 43.0% who knew their status, 86.6% were receiving ART, and among those receiving ART, 80.4% had a suppressed viral load. Under half of both resident and non‐resident populations with HIV knew their status, falling far short of the first of the 90‐90‐90 targets. However, among resident populations, the proportion of those who knew their status being on ART and the proportion of those on ART who were suppressed approached the second and third targets of 90%. In contrast, though almost all of the non‐resident population members who knew their status were on ART, only 64% of those on ART were suppressed. Figure 3 compares the marginal proportions of resident and non‐resident populations in each stage of the continuum of care to the 90‐90‐90 targets and highlights the disparity in the overall proportion suppressed between resident and non‐resident groups (31% (95% CI: 25, 37) vs. 21% (95% CI: 10, 33) respectively).

**Table 3:** Estimated percentage of people living with HIV meeting each of the 90‐90‐90 targets for the overall, resident and non‐resident populations in selected cross‐border areas in East Africa, 2016

	Overall (N = 576)		Resident (n = 460)		Non‐resident (n = 116)	
	Population %	95% CI	Population %	95% CI	Population %	95% CI
First 90: Among those with HIV, knowledge of status	43.0	38.2, 47.8	44.0	38.6, 49.4	38.9	26.9, 50.9
Second 90: Of those who know their status, on ART	86.6	82.2, 91.1	84.5	79.3, 89.8	96.2	93.2, 99.1
Third 90: Of those on ART, virologically suppressed	80.4	72.5, 88.4	83.9	74.5, 93.2	64.0	47.3, 80.8

ART, antiretroviral therapy.

### 3.3 Relationship between the 90‐90‐90 targets and participant characteristics

Overall, the strongest risk factors for having a detectable viral load were being a non‐resident of the cross border area (compared with residents of the area), being a young woman between the ages of 18 and 24 (compared with being an older woman), and being recruited at a commercial venue (e.g. market, salon), outdoor venue (e.g. sex worker street, beach, park), or other type of venue (e.g. truck stop, railway station), compared with being recruited at a bar, pub or restaurant (Table 4). Workers at venues were less likely to have a detectable viral load than patrons at venues and people working in jobs related to the fishing industry were less likely to have a detectable viral load than other participants. Associations between characteristics and individual steps of engagement in the cascade were more nuanced. For example, workers at venues were more likely to know their status (PR: 1.36, 95% CI: 1.10, 1.67) and to be virally suppressed, given that they were on ART, (PR: 1.23; 95% CI: 1.00, 1.51) than other respondents, though being a worker at a venue was not associated with being on ART (PR: 1.02; 95% CI: 0.93, 1.12). Moreover, working in a job related to the fishing industry was associated with a higher probability of knowing one's status (PR: 1.74; 95% CI: 1.41, 2.14), but was not strongly associated with being on ART (PR: 1.08; 95% CI: 0.95, 1.22), or viral suppression, conditional on being on ART (PR: 0.90; 95% CI: 0.73, 1.11).

**Table 4:** Prevalence ratios describing the associations between individual‐ and venue‐level characteristics and achievement of the 90‐90‐90 goals among people in the East Africa Cross Border Integrated Health Study, 2016

	First 90: Among those with HIV, knowledge of status		Second 90: Of those who know their status, on ART		Third 90: Of those on ART, virally suppressed		Overall probability of having a detectable viral load	
	PR	95% CI	PR	95% CI	PR	95% CI	PR	95% CI
Population type								
Resident	1		1		1		1	
Non‐resident	0.88	0.63, 1.24	1.14	1.06, 1.22	0.76	0.58, 1.01	1.15	0.96, 1.37
Gender								
Male	1		1				1	
Female	1.16	0.96, 1.40	0.97	0.88, 1.08	1.14	0.95, 1.36	0.91	0.77, 1.08
Age								
15 to 29	1		1		1		1	
30 to 39	0.71	0.57, 0.87	1.03	0.49, 2.17	1.59	0.32, 7.82	0.84	0.68, 1.02
40+	0.71	0.55, 0.91	0.36	0.12, 1.03	2.48	0.57, 10.77	0.85	0.68, 1.07
Received cash for sex in past 12 months								
No	1		1		1		1	
Yes	1.20	0.92, 1.57	0.93	0.81, 1.08	1.01	0.77, 1.33	0.92	0.71, 1.20
Works in job related to the fishing industry								
No	1		1		1		1	
Yes	1.74	1.41, 2.14	1.08	0.95, 1.22	0.90	0.73, 1.11	0.78	0.56, 1.08
Long‐distance truck drivers								
Other men	1		1					
Truck drivers	1.22	0.50, 2.97	0.93	0.59, 1.49	NA		NA	
Female workers at venues								
Other women	1		1		1		1	
Female worker	1.13	0.85, 1.49	1.03	0.90, 1.17	1.17	0.95, 1.45	0.85	0.65, 1.10
Young women								
Women 25 and older	1		1		1		1	
Women 15 to 24	0.40	0.26, 0.62	0.90	0.69, 1.17	1.09	0.87, 1.36	1.37	1.10, 1.71
Employment								
Employed	1		1		1		1	
Unemployed	0.88	0.64, 1.21	0.98	0.86, 1.13	1.00	0.76, 1.32	1.13	0.95, 1.34
Paid cash for sex in past 12 months								
No	1		1		1		1	
Yes	0.88	0.63, 1.24	1.00	0.86, 1.17	1.22	1.06, 1.40	0.93	0.66, 1.30
Currently married or living with a partner								
No	1		1		1		1	
Yes	0.99	0.80, 1.22	1.13	1.02, 1.26	0.93	0.77, 1.12	0.96	0.79, 1.16
Education								
Less than primary	1		1		1		1	
Completed primary	1.01	0.77, 1.33	0.82	0.39, 1.71	1.52	0.81, 2.86	1.05	0.86, 1.29
Completed secondary	0.96	0.72, 1.30	0.50	0.26, 0.97	2.22	0.77, 6.37	0.97	0.73, 1.29
More than secondary	0.80	0.49, 1.31	1.00	0.78, 1.28	NA		1.17	0.95, 1.43
Type of venue where recruited								
Bar/pub/restaurant	1		1		1		1	
Hotel/guest house/lodge	0.97	0.66, 1.42	1.45	0.62, 3.39	0.62	0.14, 2.83	0.71	0.39, 1.29
Nightclub/disco/brothel	0.97	0.41, 2.31	2.92	1.52, 5.61	1.57	0.71, 3.47	0.84	0.41, 1.71
Commercial venues^a^	0.83	0.63, 1.09	0.89	0.50, 1.58	0.81	0.42, 1.55	1.54	1.01, 2.35
Outside venues^b^	0.48	0.29, 0.80	1.93	0.76, 4.89	0.30	0.05, 1.73	1.76	0.98, 3.16
Other^c^	0.72	0.49, 1.07	0.97	0.86, 1.10	0.27	0.09, 0.80	1.28	0.49, 3.32
Time spent away from primary residence in past year								
Less than one month	1		1		1		1	
One month or more	0.85	0.65, 1.11	1.05	0.92, 1.21	0.98	0.76, 1.27	1.08	0.84, 1.38
Type of respondent								
Patrons at venues	1		1		1		1	
Workers at venues	1.36	1.10, 1.67	1.02	0.93, 1.12	1.23	1.00, 1.51	0.76	0.62, 0.94
Type of site where recruited								
Land site	1		1		1		1	
Lake site	1.68	1.33, 2.12	1.07	0.97, 1.19	0.96	0.80, 1.16	0.89	0.74, 1.06
Visited more than one venue on day of recruitment	0.95	0.74, 1.23	1.04	0.94, 1.14	0.96	0.79, 1.17	1.03	0.89, 1.21
Recruited at a venue where people have sex on site	0.95	0.73, 1.22	0.99	0.89, 1.10	1.10	0.91, 1.34	0.98	0.83, 1.16

PR, Prevalence ratio; CI, confidence interval; ART, antiretroviral therapy

^a^Commercial venues included markets, hair salons, shops, cinemas, recreation and game centres, and schools; ^b^Outdoor venues included beaches, parks, construction sites and streets; ^c^Other venues included truck stops, railway/lorry stations and other types of venues

## 4 Discussion

People with HIV working or socializing in East Africa cross‐border areas were unlikely to know their HIV status, although those who knew their HIV status were likely to be on treatment and suppressed: while only 43% knew their HIV‐positive status, 84% of people who knew their status were on ART and 80% of those on ART had a suppressed viral load. Both resident and non‐resident populations who knew their status were likely to be on treatment, with a notable 96% of non‐resident individuals who knew their status on treatment, suggesting that HIV care and treatment programmes are successfully starting members of this important population on treatment. However, only 64% of non‐resident individuals on treatment were suppressed, which could point to gaps in continuity of care, poor adherence or antiretroviral resistance among populations spending time outside their areas of residence in East Africa.

Cross‐border areas in East Africa are approaching the UNAIDS 90‐90‐90 goals for the proportion on ART and the proportion virally suppressed, but they fall well short of the target for the proportion of people living with HIV who know their status. These results suggest that East Africa cross border areas may be falling behind other areas in the region; UNAIDS reports that only 76% of adults with HIV between the ages of 15 and 49 in Eastern and Southern Africa knew their status as of 2017, that 60% were on ART, and that 50% were virally suppressed 25. Furthermore, the estimated proportion of people living with HIV who knew their status was lower in the selected cross‐border areas (43%) than in the baseline results from the SEARCH trial conducted in rural Kenyan and Ugandan communities in 2015 (65%) 10, the Uganda Population‐based HIV Impact Assessment conducted in 2016 to 2017 (73%) 26, or the Tanzania Population‐based HIV Impact Assessment conducted in 2016 to 2017 (52%)^27^.

Many factors may contribute to the low proportion of people with HIV who knew their status in cross‐border areas. First, cross‐border areas contain mobile groups, including truck drivers and traders, who may be missed by routine facility‐based services or community‐based testing focused on residents who spend most of their time in the area. In addition, HIV testing during regular health facility hours may be incompatible with full‐time employment, farming or family responsibilities 28. Outreach testing in public places or home‐based testing may be more effective than health facility‐based testing 12, 29, 30, 31, specifically for the mobile and migrant populations found in cross‐border areas. Furthermore, the majority of respondents (60%) who were HIV positive but did not know their status reported taking an HIV test within the previous 12 months. This high prevalence of HIV testing among people who did not know their status suggests that increasing the proportion of people living with HIV who know their status will require frequent retesting of people previously testing negative for HIV, which may be more feasible if testing is conducted outside the health facility or using home‐based testing.

Despite the low proportion of people with HIV who knew their status, people who did know their status were likely to be on treatment, pointing to successful scale‐up of HIV treatment programmes in East Africa. The CBIHS was conducted in 2016, a year when treatment guidelines in East Africa were shifting from CD4 cell count‐based thresholds for treatment initiation to a test and treat strategy. As immediate treatment continues to be normalized throughout the region, the proportion of those who know their status who are on treatment is expected to increase even further.

Outcomes measured in CBIHS in cross‐border areas may differ from outcomes measured in other studies due to differences in the sampling and recruitment techniques. For example, we sampled from people socializing in public places (“venues”), with the goal of recruiting a cross‐section of people who could be found in a cross‐border area at a given point in time. In contrast, other studies often employ household surveys to recruit residents of the selected communities. Because cross‐border sites are both home to and frequented by mobile populations, sampling both resident and non‐resident populations from public places using the PLACE sampling methodology was an important strength of this study. Cross‐border areas are important mixing grounds between resident populations and non‐resident populations travelling through the site or visiting the site for work, commerce or socializing. Such mixing between population subgroups provides important opportunities for HIV transmission 32, 33, 34, 35, 36. Accordingly, “knowing the epidemic” in a cross‐border area involves measuring viral suppression among all people who can be found in the site, and the prevention of HIV transmission must include HIV care and treatment for both resident and non‐resident groups.

Furthermore, cross‐border areas are places where behavioural determinants of transmission such as new sexual partnership rates and sexual mixing may be higher than in other areas. Many public places in cross‐border areas (48% of those sampled for verification) offer opportunities for sex on site and, therefore, direct opportunities for HIV transmission 37, 38. Venues with sex on site included not only brothels and hotels, but also bars, restaurants and nightclubs. Using the PLACE method to recruit people socializing at such venues allowed inclusion of groups at highest risk for transmitting HIV infection and those traditionally missed by household surveys, including the cross‐border priority populations of female sex workers, fisher folk and long distance truck drivers 37. Suboptimal services for people living with HIV, coupled with increased mixing and high partnership rates, means that cross‐border areas may be important drivers of the spread of the HIV epidemic across East Africa.

This study has several limitations. The non‐resident population described here is likely a heterogeneous group of individuals that included long‐term visitors to the site, daily commuters to the site for work or commerce or residents of neighbouring communities. The specific composition of this group in each cross border area may have an impact on the effectiveness of any intervention aimed at improving viral suppression. Moreover, the concept of “residence” itself is vague and may have implied different definitions to different respondents. In addition, some individuals refused the HIV test. We accounted for informative refusals of the HIV test among those who participated in the survey by up‐weighting those who agreed to be tested to represent all participants who responded to the survey, conditional on covariates. However, these weights did not account for any systematic bias that may have occurred if HIV‐positive individuals who were sampled simply refused to participate in the survey altogether.

## 5 Conclusions

Despite these limitations, this study represents the most complete information available on the HIV care and treatment cascade in strategically important cross‐border areas in East Africa. Findings suggest that people with HIV who can be found in cross‐border areas are unlikely to know their status. Moreover, people travelling outside their area of residence may face serious barriers to maintaining viral suppression even after starting treatment. These barriers could include lack of knowledge about where to get ART while away from home, cost of obtaining ART when travelling outside one's country of residence, or suboptimal outcomes due to switching regimens when obtaining ART in different countries 19.

Halting the HIV epidemic in East Africa requires preventing transmission of HIV from people infected with HIV to their uninfected partners. Improving viral suppression among populations as they travel away from home may be an efficient means to reduce HIV transmission in cross‐border areas and beyond.

## Competing interests

The other authors declare no conflicts of interest.

## Authors’ contribution

JKE designed the study, oversaw data collection, performed the analysis and wrote the manuscript. PA, EAB and JTO were involved in designing and conducting the study and revised the manuscript. FS, GM and MM were involved in study design, overseeing data collection, analysis and revision of the manuscript. AV participated in data analysis and revised the manuscript. SS was involved in study design and revised the manuscript.

## Supporting information


**Appendix S1.** Sampling details.
