# Supplementary material for: The HIV care continuum among resident and non‐resident populations found in venues in East Africa cross‐border areas
Source: J Int AIDS Soc. 2019 Jan 24;22(1):e25226. doi: 10.1002/jia2.25226 (PMC6344908; doi:10.1002/jia2.25226)
Supplement: Supplementary file 1 — Appendix S1. Sampling details. [file JIA2-22-e25226-s001.pdf]

APPENDIX

Sampling details

Sampling for Venue Verification (Step 2)
Venues reported by community informants were stratified into top, high, medium, and low priority strata based on the information reported by the community informants. In cross border areas with more than 100 venues reported, all top priority venues were visited, along with random samples of 35 high priority venues, 35 medium priority venues, and 20 low priority venues. To select venues for verification from the complete list of venues, venues were sorted by frequency with which the presence of men who have sex with men, injection drug users, and female sex workers were reported; and also by frequency of reported sex on site and the total number of times the venue was mentioned. The 10 venues highest on the sorted list were the top priority venues, with each subsequent one-third of venues assigned as high, medium and lower priority. The number of venues sampled for verification was halved in Muhuru Bay, Kenya; Kirongwe, Tanzania; Sio Port/Port Victoria, Kenya; and Majanji, Uganda due to initial plans to pool data across some of these sites.

Sampling for Patron and Worker Interviews (Step 3)
All top priority venues that were found and operational in step 3 were sampled for bio-behavioural interviews, along with a stratified random sample of high, medium, and low priority venues. At venues sampled for individual bio-behavioural interviews, the target numbers of individuals recruited per venue were determined as follows: If fewer than 20 were present at a sampled venue, all patrons and 3 workers were targeted for recruitment. If 20 to 50 people were present, targets were 3 workers, 15 male patrons, and 15 female patrons, and at larger venues, targets were set at 6 workers, 25 male patrons, and 25 female patrons. The random sample was drawn by conceptually drawing a large “X” through the venue and approaching potential respondents at predetermined points along the X. For each site, field coordinators provided estimated participation rates for male and female patrons and workers to allow for calculation of sampling probabilities.

Defining steps of the HIV care continuum

We identified individuals in each step of the HIV care continuum using responses reported on the behavioral survey, the HIV test, and viral load quantification from dried blood spots. Details are provided below. Recall that analyses in this manuscript are limited to people living with HIV. A person was considered to be living with HIV if he or she
   a. Agreed to the HIV test and tested positive; or
   b. Did not agree to the HIV test but reported previously testing positive

1. *Knows status:* A person living with HIV could have been considered to know his or her status in 2 ways:
   a. If he agreed to the HIV test, tested positive, and reported i) previously taking an HIV test, ii) receiving the result, and iii) testing positive for the previous HIV test
   b. If he did not agree to the HIV test but reported i) previously taking an HIV test, ii) receiving the result, and iii) testing positive for the previous HIV test
2. *On treatment:* A person living with HIV was considered to be on treatment if
   a. If he agreed to the HIV test, tested positive, and answered YES to the following question; or
   b. If he did not agree to the HIV test but reported i) previously taking an HIV test, ii) receiving the result, and iii) testing positive for the previous HIV test and answered YES to the following question
   c. QUESTION: Are you currently taking antiretroviral drugs (ART) to treat an HIV infection?
3. *Suppressed viral load:*
   a. A person living with HIV was considered to have a suppressed viral load if he or she agreed to the HIV test, tested positive, agreed to provide a dried blood spot, and viral load was below 1000 copies/mL
   b. A person living with HIV was considered NOT to have a suppressed viral load if he or she agreed to the HIV test, tested positive, agreed to provide a dried blood spot, and viral load was above 1000 copies/mL
   c. Otherwise, the indicator of viral suppression was missing for that person. We accounted for this missing data using inverse probability weights, as described in the methods section and below.

Details about inverse probability weights

For measures related to knowing one's status and initiation of ART, the final weight was the product of the sampling weight and the participation weight. Briefly, the sampling weight was 1 divided by the probability of being sampled for the study, as determined by the study design. The participation weight was 1 divided by the probability of participating in the HIV test conditional on informative covariates. For measures related to viral suppression, this weight was multiplied by the inverse probability of having a viral load measurement, conditional on the same set of covariates. Covariates included, age, sex, country of origin, key population status, resident or non-resident status, and cross border area. The denominators of the weights for participation and viral load measurements were estimated using multivariable logistic regression models. In these models, continuous variables were modeled flexibly using restricted quadratic splines [19] and categorical variables were modeled using indicator variables for each category.

All participants who declined the HIV test but reported being HIV-positive were missing information on viral suppression ($n = 102$). Additionally, 84 participants who tested positive for HIV during the study did not have a viral load measurement because they either refused to provide dried blood spots or the sample was not viable. Overall, 68% of resident individuals and 65% of non-resident individuals were missing viral load information .Under the assumption that viral load was independent of HIV test refusal and sample transport issues conditional on measured covariates, we used a third set of weights to reweight participants with a viral load measurement to represent all participants with HIV when estimating the proportion virologically suppressed. The viral load weights were 1 divided by the predicted probability of having a viral load measurement conditional on age, sex, country of origin, key population status, and cross border area, and, given that the missing data was monotonic [32], the final weight used to estimate viral suppression was the product of the sampling, participation, and viral load weights.
